# Ultrasound in Cardiovascular Risk Prediction: Don't Forget the Plaque!

**DOI:** 10.1161/JAHA.113.000180

**Published:** 2013-04-24

**Authors:** Vijay Nambi, Gerd Brunner, Christie M. Ballantyne

**Affiliations:** 1 Department of Medicine, Baylor College of Medicine, Houston, TX (V.N., G.B., C.M.B.); 2 The Methodist DeBakey Heart and Vascular Center, The Methodist Hospital, Houston, TX (V.N., G.B., C.M.B.); 3 Michael E. DeBakey Veterans Affairs Medical Center, Houston, TX (V.N.); 4 Ben Taub General Hospital, Houston, TX (V.N.)

**Keywords:** Editorials, carotid arteries, intima–media thickness, plaque, ultrasound

## Introduction

Ultrasound‐based cardiovascular risk stratification has almost come full circle. Since its early description by Pignoli et al,^1^ carotid intima–media thickness (CIMT) measures have been correlated with all traditional risk factors and cardiovascular diseases (CVD)^2^ and have begun to find use in clinical trials aimed at evaluating antiatherosclerotic therapy (as a surrogate marker). Despite recent analyses from studies such as the Atherosclerosis Risk in Communities (ARIC) study,^3–4^ which demonstrated the value of ultrasound in coronary heart disease (CHD) risk prediction, other analyses such as a recent meta‐analysis^5–6^ have suggested that the improvement offered by CIMT was small and unlikely to be of clinical importance. However, the meta‐analysis did not include plaque data and has resulted in some confusion related to the value of ultrasound‐based subclinical atherosclerosis estimation and its role in CVD risk stratification. The analysis of the Multi‐Ethnic Study of Atherosclerosis (MESA) by Polak et al,^7^ presented in this issue of *JAHA*—*Journal of the American Heart Association*, along with other recent reports that have incorporated plaque information, such as from the Three‐City Study,^8^ have confirmed the importance of using plaque assessment for ultrasound‐aided CVD risk assessment. The current analysis further adds to the literature by examining several plaque definitions and reporting on their association with and value in risk prediction. Overall, the study showed value in CHD prediction but not in stroke, which, we agree with the authors, was likely related to the small number of stroke events that have accumulated so far in MESA, given previous results from other studies such as the ARIC study.^9^

The “intima–media thickness (IMT) complex” includes both the intima and the media (predominantly smooth muscle); in states of health, approximately 97.5% of the IMT complex is made of the media, whereas in the presence of atherosclerotic disease, although the intimal contribution to the IMT complex is relatively higher, an estimated 80% of the IMT complex still is formed by the media.^10^ Given that atherosclerosis is a subintimal process, the measurement of IMT is in a way a surrogate of a surrogate. Plaque on the other hand reflects focal increases in the thickness of the arterial wall and is more likely to represent definite areas of atherosclerosis rather than medial hypertrophy. Therefore, relative to IMT, plaque may have a stronger association with CHD events, as demonstrated in this study.

The importance of plaque has been shown in several studies. In the ARIC study,^3–4^ we have shown that information about plaque presence in addition to CIMT added predictive value irrespective of the CIMT category (<25th percentile, 25th to 75th percentile, and >75th percentile) and that its value was greater in women. Similarly, the Framingham Offspring Study^11^ suggested that information about plaque presence (defined as an IMT in the internal carotid artery >1.5 mm) was associated with improved prediction of CVD risk (Table), whereas investigators from the Three‐City Study^8^ showed that plaque but not CIMT added to CVD risk prediction. The investigators in the Three‐City Study further showed that as the number of sites with plaque increased, so did the hazards for incident CVD events.^8^ Finally, a meta‐analysis of 11 population‐based studies (54 336 subjects) suggested that plaque was more strongly associated with incident CHD when compared to CIMT.^12^ On the other hand, results related to CIMT alone have been mixed. A recent prospective European study (the Carotid Intima‐Media Thickness [CIMT] and IMT‐Progression as Predictors of Vascular Events in a High Risk European Population [IMPROVE] study) suggested that CIMT adds to CHD risk prediction,^13^ whereas other studies such as the Carotid Atherosclerosis Progression Study (CAPS)^5^ and a meta‐analysis^14^ (14 CIMT studies, 45 828 subjects) suggested limited value for CIMT in CHD risk prediction and the Rotterdam Study^15^ showed mixed results in that CIMT added value in the cardiovascular risk stratification of older women, but not of older men. While limitations in each of these analyses merit consideration, overall, in general plaque information consistently seems to add to traditional risk factors in CHD risk prediction.

**Table 1.** Epidemiological Studies Reporting on the Use of CIMT and Carotid Artery Plaque in the Prediction of CHD and CVD Risk

	ARIC^4^	CAPS^5^	Framingham Offspring^11^	MESA^7^
Patients, n	13 145	4904	2965	6562
Mean follow‐up, y	15.1	8.5	7.2	7.8
Events				
MI, n	867	73	NR	NR
CHD deaths/deaths, n	159	72	NR	NR
Other CVD events, n	Silent MI: 98; revasc.: 688	AP or MI: 271	Stroke: 74; CHD: 152; PAD: 26; CHF: 45	Stroke: 139; incident CVD: 515
Ultrasound data				
IMT segments	All (mean)	ICA, CCA, bifurcation	Max. ICA, mean CCA	Mean ICA, max. ICA
Plaque presence	Yes	Yes	Yes	Yes
AUC with CIMT/plaque measurements				
AUC (TRF)	0.742	0.719	0.748	0.743
AUC (CIMT+TRF)	0.750	0.724	0.751	0.750
AUC	0.755*	0.722*	0.758*	—
AUC (plaque+TRF)	0.751	NR	0.762	0.750
NRI with CIMT/plaque measurements added to TRF				
NRI, %	9.9*	NR	7.3*	5.0*
NRI (CCA), %	16.2	–1.4	0	NR
NRI (ICA), %	NR	1.6	7.6	7.0*/6.8*

CIMT indicates carotid intima–media thickness; CHD, coronary heart disease; CVD, cardiovascular disease; ARIC, Atherosclerosis Risk in Communities; CAPS, Carotid Atherosclerosis Progression Study; MESA, Multi‐Ethnic Study of Atherosclerosis; MI, myocardial infarction; NR, not reported; revasc., coronary revascularizations; AP, angina pectoris; PAD, peripheral arterial disease; CHF, congestive heart failure; ICA, internal carotid artery, CCA, common carotid artery; AUC, area under curve; TRF, traditional risk factors; NRI, net reclassification index.

* CIMT+plaque+TRF.

* ICA+TRF.

* CIMT+plaque.

* Plaque.

* Mean of the maximum IMT measured in ICA (based on CHD events).

* Maximum IMT measured in ICA (based on CHD events).

The analyses presented by Polak et al^7^ in this issue of *JAHA* has long been awaited since MESA reported on CIMT compared with coronary calcium score and other measures of atherosclerosis without examining the benefit of information about plaque.^16^ This analysis, along with the preponderance of evidence from other large epidemiological studies, affirm the class IIA indication given to ultrasound‐based CIMT/plaque detection in the CHD risk prediction guidelines published by the American College of Cardiology and American Heart Association.^17^

However, when considering the value of ultrasound as a tool for assessing atherosclerosis risk, one must be cognizant of the various advantages and disadvantages. One of the limitations of ultrasound is image quality, which depends highly on the sonographer‘s ability to provide a comprehensive scan using appropriate standardized angles, etc. Tests such as the coronary calcium score, on the other hand, are fairly automated and easy to perform. Patient body habitus can similarly have a greater impact on ultrasound images relative to other imaging modalities. Finally, since CIMT measures are exacting, small changes (which may occur with minor changes in the angle of imaging) can have an impact on the measured value and hence the interpretation of the test. The advantages of ultrasound scanning are several: there are no major side effects to the test (minimal heating of tissue is possible), scans can be done on portable devices, and the overall acquisition time is fast, which offers the possibility of higher throughput, lower cost, and relative safety.

One significant technological advance (since epidemiological studies such as ARIC and MESA were performed) that will likely further enhance the value of ultrasound is the advent of 3D ultrasound imaging, which will allow a more quantitative approach in assessing the burden of atherosclerosis. Several groups have described the value of being able to assess the number of plaques and the plaque areas in the ultrasound images of the carotids;^8,18^ however, these techniques would be more effective if they were more quantitative and less labor intensive. The advent of 3D ultrasound may now offer a relatively easier method for quantifying atherosclerotic plaque. Given that the mere presence of plaque adds to CHD risk prediction, one may hypothesize that plaque volumes will be of great benefit to risk prediction. Existing data have already suggested that quantification with plaque scores (formulated on the basis of number of plaques) and plaque area show value (ie, more plaques are associated with worse outcomes).^7–8^ The early studies of 3D ultrasound plaque volumes measured with commercially available scanners have been promising and have shown that plaque volumes are highly associated with coronary calcium scores^19^ and, in a small study, had a good negative predictive value for CHD.^20^

The need to improve CVD risk stratification remains, since the majority of CVD events occur in patients classified in the “low” and “intermediate” risk groups. As newer therapies are identified, improved methods to identify higher risk will also be necessary. An accurate, cost effective, and safe method will clearly be desirable. The current study published in this issue of *JAHA*^7^ along with other recent studies suggest that ultrasound‐based plaque estimation remains an attractive option for assessing atherosclerosis and its associated risk.

## References

[1] Pignoli P Tremoli E Poli A Oreste P Paoletti R Intimal plus medial thickness of the arterial wall: a direct measurement with ultrasound imaging. Circulation. 1986; 74:1399-1406 3536154 10.1161/01.cir.74.6.1399

[2] Chambless LE Heiss G Folsom AR Rosamond W Szklo M Sharrett AR Clegg LX Association of coronary heart disease incidence with carotid arterial wall thickness and major risk factors: the Atherosclerosis Risk in Communities (ARIC) Study, 1987–1993. Am J Epidemiol. 1997; 146:483-494 9290509 10.1093/oxfordjournals.aje.a009302

[3] Nambi V Chambless L He M Folsom AR Mosley T Boerwinkle E Ballantyne CM Common carotid artery intima‐media thickness is as good as carotid intima‐media thickness of all carotid artery segments in improving prediction of coronary heart disease risk in the Atherosclerosis Risk in Communities (ARIC) study. Eur Heart J. 2011; 33:183-190 21666250 10.1093/eurheartj/ehr192 PMC3258447

[4] Nambi V Chambless L Folsom AR He M Hu Y Mosley T Volcik K Boerwinkle E Ballantyne CM Carotid intima‐media thickness and presence or absence of plaque improves prediction of coronary heart disease risk: the ARIC (Atherosclerosis Risk In Communities) study. J Am Coll Cardiol. 2010; 55:1600-1607 20378078 10.1016/j.jacc.2009.11.075 PMC2862308

[5] Lorenz MW Schaefer C Steinmetz H Sitzer M Is carotid intima media thickness useful for individual prediction of cardiovascular risk? Ten‐year results from the Carotid Atherosclerosis Progression Study (CAPS). Eur Heart J. 2010; 31:2041-2048 20530503 10.1093/eurheartj/ehq189

[6] Lorenz MW Polak JF Kavousi M Mathiesen EB Volzke H Tuomainen TP Sander D Plichart M Catapano AL Robertson CM Kiechl S Rundek T Desvarieux M Lind L Schmid C DasMahapatra P Gao L Ziegelbauer K Bots ML Thompson SG Carotid intima‐media thickness progression to predict cardiovascular events in the general population (the PROG‐IMT collaborative project): a meta‐analysis of individual participant data. Lancet. 2012; 379:2053-2062 22541275 10.1016/S0140-6736(12)60441-3 PMC3918517

[7] Polak JF Szklo M Kronmal RA Burke GL Shea S Zavodni AEH O'Leary DH The value of carotid artery plaque and intima‐media thickness for incident cardiovascular disease: the Multi‐Ethnic Study of Atherosclerosis. J Am Heart Assoc. 2013; 2:e00008710.1161/JAHA.113.000087 23568342 10.1161/JAHA.113.000087 PMC3647272

[8] Plichart M Celermajer DS Zureik M Helmer C Jouven X Ritchie K Tzourio C Ducimetiere P Empana JP Carotid intima‐media thickness in plaque‐free site, carotid plaques and coronary heart disease risk prediction in older adults. The Three‐City Study. Atherosclerosis. 2011; 219:917-924 22005196 10.1016/j.atherosclerosis.2011.09.024

[9] Ohira T Shahar E Iso H Chambless LE Rosamond WD Sharrett AR Folsom AR Carotid artery wall thickness and risk of stroke subtypes: the Atherosclerosis Risk in Communities study. Stroke. 2011; 42:397-403 21164133 10.1161/STROKEAHA.110.592261 PMC3026889

[10] Spence JD Ultrasound measurement of carotid plaque as a surrogate outcome for coronary artery disease. Am J Cardiol. 2002; 89:10B-15B‐ 10.1016/s0002-9149(01)02327-x 11879661

[11] Polak JF Pencina MJ Pencina KM O'Donnell CJ Wolf PA D'Agostino RB Sr Carotid‐wall intima‐media thickness and cardiovascular events. N Engl J Med. 2011; 365:213-221 21774709 10.1056/NEJMoa1012592 PMC3153949

[12] Inaba Y Chen JA Bergmann SR Carotid plaque, compared with carotid intima‐media thickness, more accurately predicts coronary artery disease events: a meta‐analysis. Atherosclerosis. 2012; 220:128-133 21764060 10.1016/j.atherosclerosis.2011.06.044

[13] Baldassarre D Hamsten A Veglia F de Faire U Humphries SE Smit AJ Giral P Kurl S Rauramaa R Mannarino E Grossi E Paoletti R Tremoli E Measurements of carotid intima‐media thickness and of interadventitia common carotid diameter improve prediction of cardiovascular events: results of the IMPROVE (Carotid Intima Media Thickness [IMT] and IMT‐Progression as Predictors of Vascular Events in a High Risk European Population) study. J Am Coll Cardiol. 2012; 60:1489-1499 22999719 10.1016/j.jacc.2012.06.034

[14] Den Ruijter HM Peters SA Anderson TJ Britton AR Dekker JM Eijkemans MJ Engstrom G Evans GW de Graaf J Grobbee DE Hedblad B Hofman A Holewijn S Ikeda A Kavousi M Kitagawa K Kitamura A Koffijberg H Lonn EM Lorenz MW Mathiesen EB Nijpels G Okazaki S O'Leary DH Polak JF Price JF Robertson C Rembold CM Rosvall M Rundek T Salonen JT Sitzer M Stehouwer CD Witteman JC Moons KG Bots ML Common carotid intima‐media thickness measurements in cardiovascular risk prediction: a meta‐analysis. JAMA. 2012; 308:796-803 22910757 10.1001/jama.2012.9630

[15] Elias‐Smale SE Kavousi M Verwoert GC Koller MT Steyerberg EW Mattace‐Raso FU Hofman A Hoeks AP Reneman RS Witteman JC Common carotid intima‐media thickness in cardiovascular risk stratification of older people: the Rotterdam Study. Eur J Prev Cardiol. 2012; 19:698-705 21697209 10.1177/1741826711414623

[16] Folsom AR Kronmal RA Detrano RC O'Leary DH Bild DE Bluemke DA Budoff MJ Liu K Shea S Szklo M Tracy RP Watson KE Burke GL Coronary artery calcification compared with carotid intima‐media thickness in the prediction of cardiovascular disease incidence: the Multi‐Ethnic Study of Atherosclerosis (MESA). Arch Intern Med. 2008; 168:1333-1339 18574091 10.1001/archinte.168.12.1333 PMC2555989

[17] Greenland P Alpert JS Beller GA Benjamin EJ Budoff MJ Fayad ZA Foster E Hlatky MA Hodgson JM Kushner FG Lauer MS Shaw LJ Smith SC Jr Taylor AJ Weintraub WS Wenger NK Jacobs AK Anderson JL Albert N Buller CE Creager MA Ettinger SM Guyton RA Halperin JL Hochman JS Nishimura R Ohman EM Page RL Stevenson WG Tarkington LG Yancy CW 2010 ACCF/AHA guideline for assessment of cardiovascular risk in asymptomatic adults: a report of the American College of Cardiology Foundation/American Heart Association Task Force on Practice Guidelines. J Am Coll Cardiol. 2010; 56:e50-e103 21144964 10.1016/j.jacc.2010.09.001

[18] Mathiesen EB Johnsen SH Wilsgaard T Bonaa KH Lochen ML Njolstad I Carotid plaque area and intima‐media thickness in prediction of first‐ever ischemic stroke: a 10‐ year follow‐up of 6584 men and women: the Tromso Study. Stroke. 2011; 42:972-978 21311059 10.1161/STROKEAHA.110.589754

[19] Sillesen H Muntendam P Adourian A Entrekin R Garcia M Falk E Fuster V Carotid plaque burden as a measure of subclinical atherosclerosis: comparison with other tests for subclinical arterial disease in the High Risk Plaque BioImage study. JACC Cardiovasc Imaging. 2012; 5:681-689 22789936 10.1016/j.jcmg.2012.03.013

[20] Johri AM Chitty DW Matangi M Malik P Mousavi P Day A Gravett M Simpson C Can carotid bulb plaque assessment rule out significant coronary artery disease? A comparison of plaque quantification by two‐ and three‐dimensional ultrasound. J Am Soc Echocardiogr. 2013; 26:86-95 23084880 10.1016/j.echo.2012.09.005

